# Transcutaneous Electrical Spinal Cord Neuromodulator (TESCoN) Improves Symptoms of Overactive Bladder

**DOI:** 10.3389/fnsys.2020.00001

**Published:** 2020-02-06

**Authors:** Evgeniy Kreydin, Hui Zhong, Kyle Latack, Shirley Ye, V. Reggie Edgerton, Parag Gad

**Affiliations:** ^1^Institute of Urology, Keck School of Medicine, University of Southern California, Los Angeles, CA, United States; ^2^Rancho Research Institute, Rancho Los Amigos National Rehabilitation Center, Downey, CA, United States; ^3^Department of Neurobiology, University of California, Los Angeles, Los Angeles, CA, United States; ^4^Department of Integrative Biology and Physiology, University of California, Los Angeles, Los Angeles, CA, United States; ^5^Department of Neurosurgery, University of California, Los Angeles, Los Angeles, CA, United States; ^6^Brain Research Institute, University of California, Los Angeles, Los Angeles, CA, United States; ^7^Institut Guttmann, Hospital de Neurorehabilitació, Institut Universitari adscrit a la Universitat Autònoma de Barcelona, Barcelona, Spain; ^8^The Centre for Neuroscience and Regenerative Medicine, Faculty of Science, University of Technology Sydney, Ultimo, NSW, Australia

**Keywords:** non-invasive spinal cord stimulation, spinal cord injury, stroke, multiple sclerosis, neurogenic bladder, over active bladder urodynamics

## Abstract

Neuromodulation is a therapeutic technique that is well-established in the treatment of idiopathic Lower urinary tract (LUT) dysfunction such as overactive bladder (OAB). We have recently developed a novel neuromodulation approach, Transcutaneous Electrical Spinal Cord Neuromodulation (TESCoN) and demonstrated its acute effects on LUT dysfunction after spinal cord injury (SCI) during urodynamic studies. We found that TESCoN can promote urinary storage and induce urinary voiding when delivered during urodynamic studies. The objective of this study was to determine whether TESCoN can retrain the spinal neural networks to induce chronic improvement in the LUT, such that positive changes can persist even in the absence of stimulation. In addition, we wished to examine the effect of TESCoN on LUT dysfunction due to multiple pathologies. To achieve this objective, 14 patients [SCI = 5, stroke = 5, multiple sclerosis (MS) = 3, and idiopathic OAB (iOAB) = 1] completed 24 sessions of TESCoN over the course of 8 weeks. Patients completed urodynamic studies before and after undergoing TESCoN therapy. Additionally, each subject completed a voiding diary and the Neurogenic Bladder Symptom Score questionnaire before and after receiving TESCoN therapy. We found that TESCoN led to decreased detrusor overactivity, improved continence, and enhanced LUT sensation across the different pathologies underlying LUT dysfunction. This study serves as a pilot in preparation for a rigorous randomized placebo-controlled trial designed to demonstrate the effect of TESCoN on LUT function in neurogenic and non-neurogenic conditions.

**New And Noteworthy:**

Non-Surgical modality to reduce incidence of urinary incontinence and improve neurogenic bladder symptom scores (NBSS) in individuals with neurogenic bladder due to spinal cord injury or stroke.

## Introduction

The lower urinary tract (LUT, consisting of the bladder and bladder outlet) serves two main roles: to store and empty urine. LUT dysfunction occurs when either storage or voiding are impaired, resulting in urinary incontinence or retention. LUT dysfunction is common in patients with neurological disease and the general population (de Groat, 1997; Jeong et al., 2010). In the case of neurological disease, LUT dysfunction occurs because the normal pathways responsible for communication between the LUT and the neural micturition centers become disrupted. While the mechanism of idiopathic LUT dysfunction is not as obvious, the nervous system is thought to be at least partially implicated in the majority of cases. LUT dysfunction has profound effects which range from endangering patients’ health [as the case of poorly managed LUT dysfunction after spinal cord injury (SCI)] to significantly impacting patients’ quality of life [as in the case of idiopathic over active bladder (iOAB) and post-stroke LUT dysfunction].

While it is often assumed that paralyzed individuals prioritize recovery of ambulation, multiple studies have demonstrated that restoration of bladder function is ranked among the top 2–3 priorities, above goals such as regaining lower extremity function (Anderson, 2004). Likewise, urinary incontinence after stroke is a well-known risk factor for long-term disability, depression and institutionalization (Panfili et al., 2017). Current therapy for LUT focuses on managing these complications without addressing the underlying cause or attempting to normalize or restore function (Stohrer et al., 2009). Urinary incontinence, frequency and urgency present across diseases such as SCI, stroke, multiple sclerosis (MS) and iOAB. While the reasons for this may vary, detrusor overactivity (or uninhibited detrusor contractions) is a common physiologic phenomenon observed in these conditions. Multiple therapies exist for correcting urinary storage function; however, they are not always suitable populations (e.g., anticholinergic medications in patients with cognitive impairment; intravesical botulinum toxin in patients at risk for retention) and they do not attempt to restore normal LUT function. On the other hand, the premise of neuromodulation is to correct the underlying neurological deficit and thus restore function to an end organ. Some neuromodulation techniques are well-established in iOAB including sacral nerve stimulation (Dasgupta et al., 2005) and percutaneous tibial nerve stimulation (Peters et al., 2010). We have recently developed a novel neuromodulation approach, Transcutaneous Electrical Spinal Cord Neuromodulation (TESCoN) a novel non-invasive neuromodulation technique to facilitate functional restoration after neurological injury (Gad et al., 2019). This modality engages the automaticity and the feedforward (Gerasimenko et al., 2017) features of the spinal neural networks to activate the intrinsic control of the spinal networks that is sufficient to enable recovery of voluntary control. We have previously demonstrated that acute TESCoN facilitates urinary storage and promotes bladder emptying in individuals with SCI during urodynamic studies (Gad et al., 2018a). Patients experienced decreased detrusor overactivity, exhibited increased bladder capacity and improved detrusor-sphincter dyssynergia when stimulation was delivered at a high frequency; on the other hand, when stimulation was delivered at a low frequency subjects demonstrated improved voiding efficiency. These changes in LUT function were only noted during active stimulation. In this study we wished to determine whether repetitive stimulation over the course of several weeks can retrain the spinal neural networks to relearn timely storage and voiding. In addition, given the similarities in storage LUT symptoms and physiologic phenomena (e.g., detrusor overactivity) across multiple conditions, we wished to expand the application of TESCoN to LUT dysfunction due to stroke, MS and iOAB. Finally, our objective was to provide a clinical assessment of the effect of TESCoN on the LUT by examining changes in voiding diaries and validated clinical questionnaire following a course of the therapy.

## Materials and Methods

### Patient Recruitment

This study was approved by the Institutional Review Board of Rancho Research Institute, the research arm of Rancho Los Amigos National Rehabilitation Center, Downey, CA, United States. All research participants signed an informed consent form before the start of the study and consented to their data being used in future publications and presentations. Five patients (four males and one female) with stable (greater than 1-year post diagnosis) SCI at T8 or above who used clean intermittent catheterization (CIC), five patients (three males and two females) with stable cortical stroke (greater than 1-year post diagnosis), three female patients with progressive MS symptoms for at least 1 year and one female patient with idiopathic OAB were recruited (Table 1). All patients experienced symptoms of urinary incontinence and sensate (i.e., non-SCI) patients reported urinary frequency and urgency.

**TABLE 1:** Table summarizing 14 patients, their pathology (*n* = 5 SCI, 5 Stroke, *n* = 3 MS and *n* = 1iOAB) location of injury, severity of injury, months post injury, current bladder management technique, LUT symptoms and current medications.

**Pt#**	**Age (yrs)**	**Gender**	**Pathology**	**Location**	**Severity**	**Months post**	**Bladder management**	**Lower urinary tract symptoms**	**Lower urinary tract medications**
P1	25–35	M	SCI	T4	AIS A	18 m	CIC	Incontinence	None
P2	25–35	F	SCI	T6	AIS A	29 m	CIC	Incontinence	Mirabegron 50 mg
P3	40–50	M	SCI	C4	AIS C	20 m	CIC	Urge Incontinence	None
P4	35–45	M	SCI	T5	AIS A	135 m	CIC	Urge Incontinence	Tolterodine LA 4 mg
P5	50–60	M	SCI	T9	AIS C	48 m	CIC	Urgency/Incontinence	Solifenacin 10 mg
P6	40–50	M	CVA	L Basal Ganglia		50 m	Volitional	Urgency/Nocturia	Tolterodine LA 4 mg
P7	40–50	M	CVA	R Basal Ganglia		36 m	Volitional	Urgency/urge incontinence	Tolterodine LA 4 mg
P8	55–65	F	CVA	L Centrum Semiovale		78 m	Volitional	Urge incontinence	Tolterodine LA 4 mg
P9	55–65	F	CVA	L Basal Ganglia		75 m	Volitional	Urge incontinence	Oxybutynin 5 mgTID
P10	55–65	M	CVA	LMCA		75 m	Volitional	Urgency/Frequency	None
P11	20–30	F	MS			48 m	Volitional	Urge incontinence	None
P12	55–65	F	MS			240 m	Volitional	Incontinence	None
P13	35–45	F	MS			24 m	Volitional	Urge incontinence	Tolterodine LA 4 mg, Tamsulosin 0.8 mg
P14	55–65	F	iOAB			48 m	Volitional	Urge Incontinence/Frequency	None

*SCI, spinal cord injury; CVA, cerebral vascular accident; MS, multiple sclerosis; CIC, clean intermittent catheterization.*

### Initial Assessment

Each patient underwent a detailed medical history and physical examination and completed an assessment of LUT symptoms using the Neurogenic Bladder Symptom Score (NBSS). A baseline urodynamic study was performed in SCI and stroke subjects according to International Continence Society (ICS) guidelines using a Goby Urodynamics System from Laborie (Ontario, Canada). In order to mimic a clinical setting where patients may not be evaluated with urodynamics prior to therapy, MS and idiopathic OAB subjects were assessed only with a detailed history and physical, a voiding diary, and the NBSS. Following the initial visit, each subject completed a 4-day voiding diary.

### Delivery of Spinal Stimulation

Stimulation was delivered using a proprietary TESCoN device (spineX, Inc.) (Gad et al., 2019). The stimulation waveform consisted of two alternating pulses of opposite polarities separated by a 1 μS delay to form a delayed biphasic waveform. The pulses consisted of a high frequency biphasic carrier pulse (10 KHz) combined with a low frequency (30 Hz) burst pulse each with a pulse width of 1 ms. Stimulation was applied using an adhesive electrode over the interspinous ligaments of T11 and L1 serving as the cathode and two adhesive electrodes over the iliac crests as the anodes. The frequencies were selected based on our previous findings demonstrating greatest reduction in incontinence and increase in bladder capacity (Gad et al., 2018a).

### Identification of Stimulation Parameters

**FIGURE 1:**
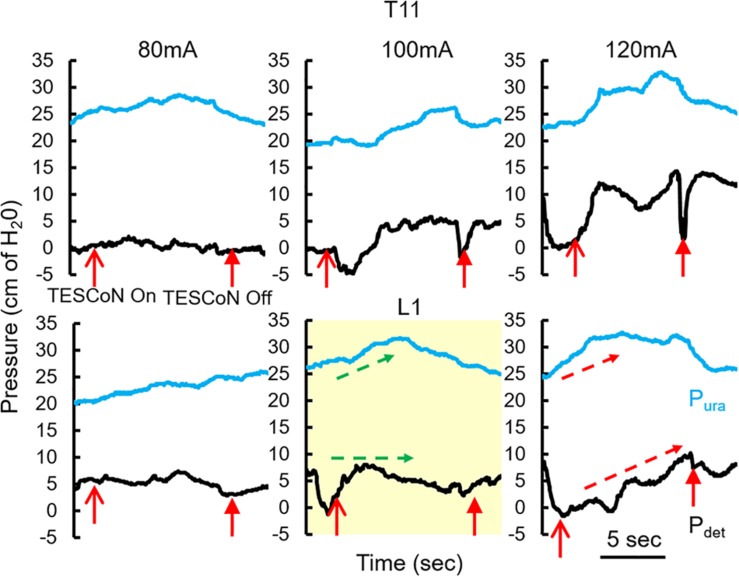
Changes in detrusor and urethral pressures with changing parameters of TESCoN. Representative patient demonstrating the protocol to identify parameters of TESCoN that generates minimal change in Pdet along with a change in Pura. In this case stimulation at L1 at 100 mA (yellow box) generated no change in Pdet while Pura increased from ∼25 to 32 cm of H20 (green arrows) between TESCoN On and TESCoN off. Note the increase in both the Pura and Pdet at L1 120 mA.

Patients with SCI and stroke underwent formal evaluation for selection of stimulation parameters as previously published (Gad et al., 2018a). In brief, a urodynamic two-port urethral catheter and a urodynamic rectal catheter were placed to measure intravesical (P_ves_), external urethral sphincter (P_ura_) and abdominal (P_abd_) pressures, respectively. Stimulation was delivered as described above. Dose response curves were constructed for each parameter with incremental increase in stimulation intensity. The stimulation intensity that generated a noticeable change in P_ura_ with little to no change in P_det_ was selected (Figure 1). This stimulation intensity did not cause any discomfort to the patients. Urodynamic studies were then performed according to ICS guidelines with concurrent TESCoN stimulation. Again, to mimic a clinical setting which precludes such an assessment, subjects with MS and idiopathic OAB were stimulated at a preselected frequency (30 Hz) and location (T11 and L1). Stimulation intensity was set as the highest current that did not cause cutaneous discomfort or cause any muscle activation in pelvic floor muscles or lower extremity muscles.

### TESCoN Therapy Course

Following the baseline evaluation, the patients were invited to return for an 8-week long course of TESCoN Subjects received stimulation for 90 min. Each subject completed three stimulation sessions a week.

### Post-stimulation Assessment

Within 1 week after the last stimulation session, SCI and stroke patients completed another clinical urodynamic study in the absence of TESCoN. All patients also completed the NBSS at this time and submitted a voiding diary starting 4 days prior to the final assessment.

### Data and Statistical Analysis

The following urodynamic variables were collected in SCI and stroke subjects: (1) Bladder capacity, (2) Voiding efficiency, (3) Maximum detrusor pressure during voiding contraction, (4) Change in urethral sphincter pressure during filling and voiding contraction, (5) volume at first sensation and (6) time between bladder capacity and beginning of voiding contraction. “ΔP_ura_ Filling” was defined as the change in pressure observed in P_ura_ during the filling cycle. “P_ura_ Baseline” was defined as the pressure in the P_ura_ prior to start of filling. “ΔP_det_ void” and “ΔP_ura_ void” were defined as the change in pressures observed in P_det_ and ΔP_ura_, respectively, between bladder capacity and voiding. The paired *t*-test was used to determine the significance of differences in urodynamic parameters, NBSS scores of participants and number of daily voids and incontinence episodes with and without TESCoN and before and after therapy.

## Results

### Urodynamic Assessment of SCI and Stroke Patients

**FIGURE 2:**
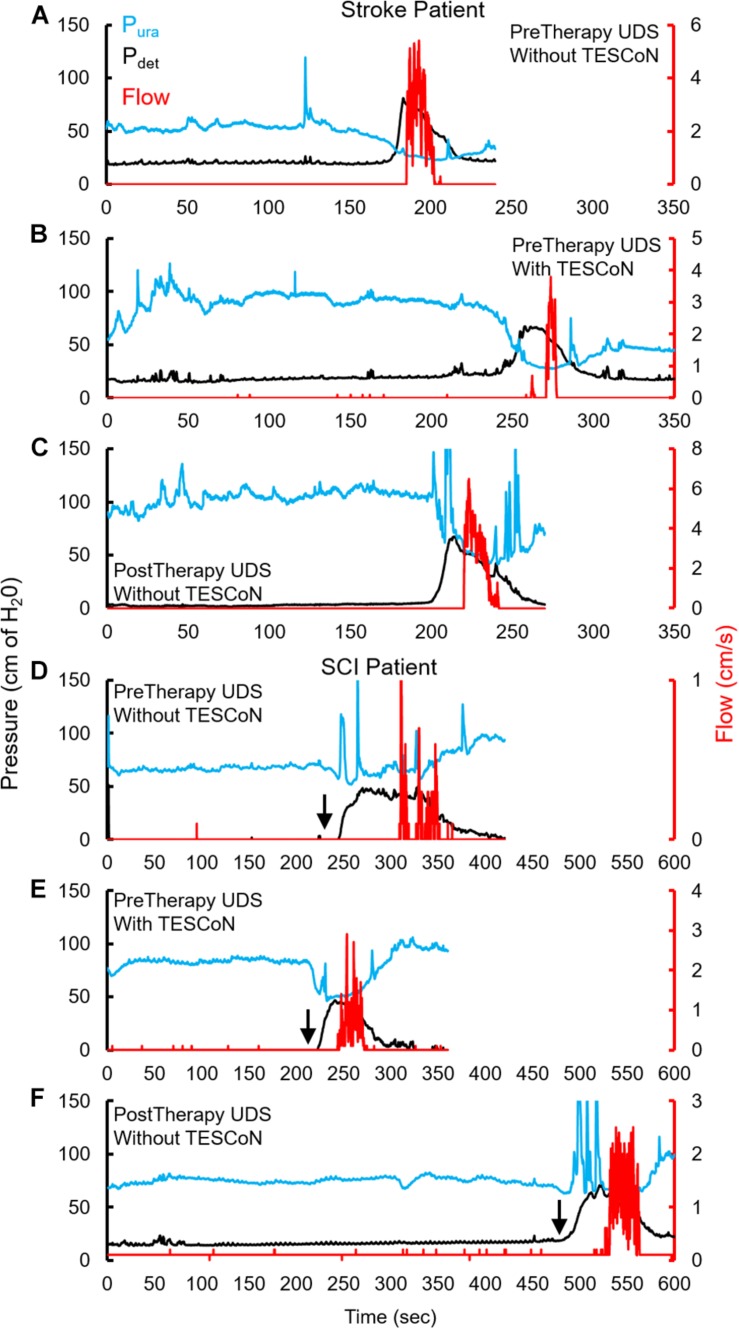
Changes in urodynamic studies without and with TESCoN. Representative urodynamic study for a stroke patient, **(A)** before therapy (PreTherapy) without and **(B)** with TESCoN and **(C)** after therapy (PostTherapy) and spinal cord injured (SCI) **(D)** before therapy (PreTherapy) without and **(E)** with TESCoN and **(F)** after therapy (PostTherapy). Note the increased bladder capacity (time prior to detrusor contraction), improved flow, improved detrusor and sphincter coordination and increase in urethral pressure during filling both with TESCoN at PreTherapy and PostTherapy (without TESCoN). Black arrow marks the occurrence of detrusor overactivity.

**FIGURE 3:**
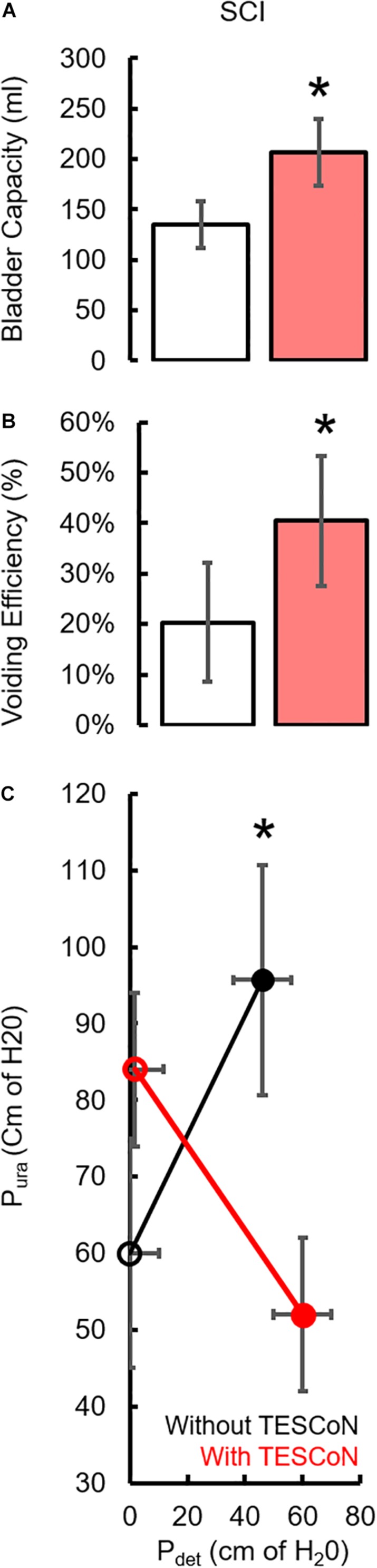
Changes in urodynamic parameters during acute stimulation in SCI subjects. Mean ±SE (*n* = 5 SCI) without (white bar) and with (red bar) acute delivery of TESCoN. **(A)** bladder capacity, **(B)** Voiding efficiency, **(C)** changes in pressure during filling vs. voiding to demonstrate the improvement in Detrusor-Sphincter Dyssynergia (DSD) without (black) and with TESCoN (red). * statistically significant from without TESCoN at *P* < *0.05.*

**FIGURE 4:**
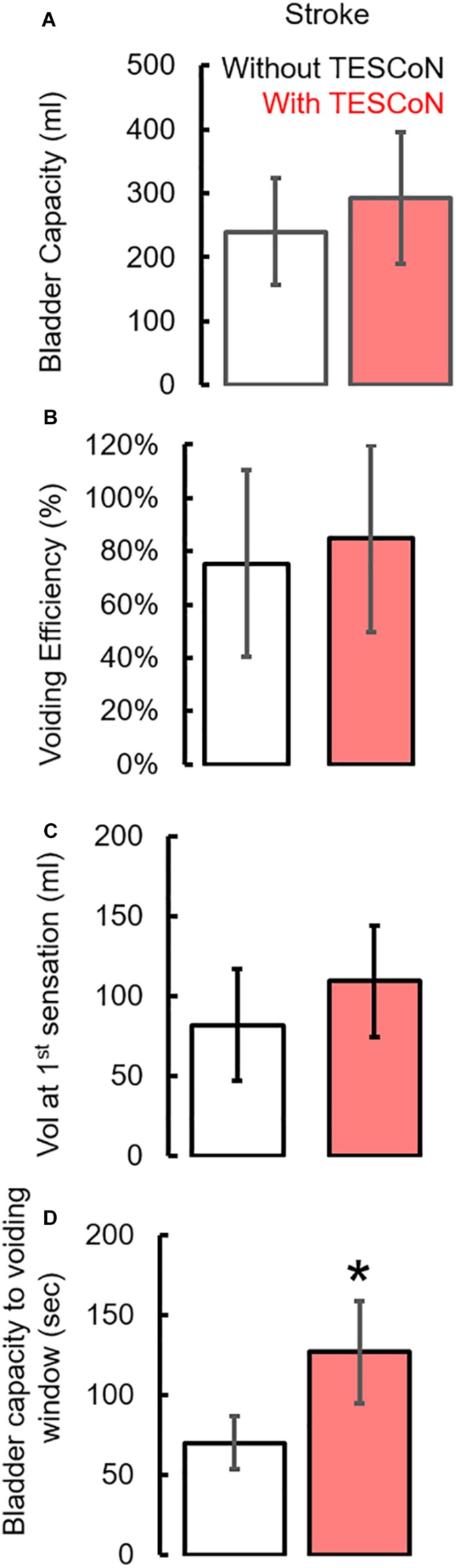
Changes in urodynamic parameters during acute stimulation in stroke subjects. Mean ±SE (*n* = 5 Stroke) **(A)** bladder capacity, **(B)** Voiding efficiency, **(C)** volume at first sensation during urodynamic study without (white bar) and with (red bar) acute TESCoN and **(D)** time window between bladder capacity and voiding in stroke patients. * statistically significant from without TESCoN at *P* < *0.05.*

**FIGURE 5:**
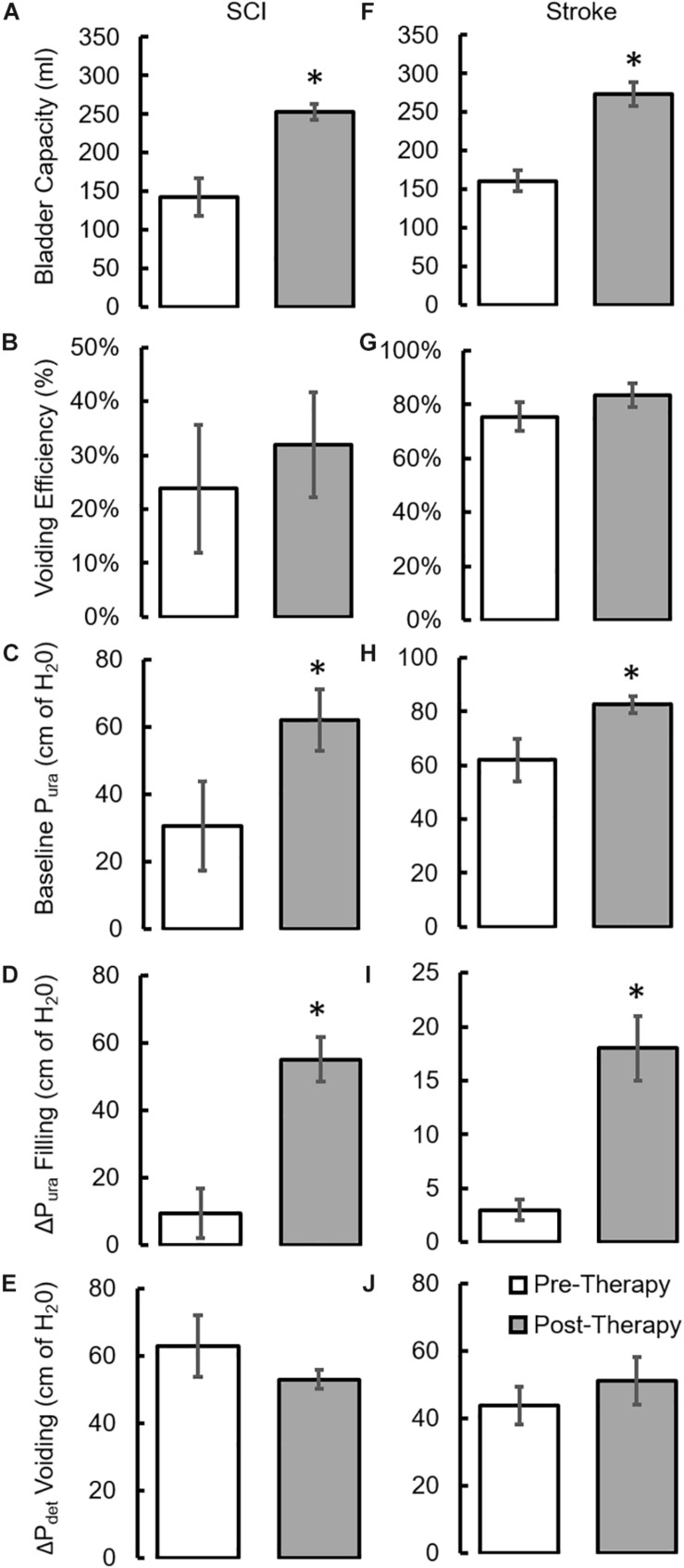
Changes in urodynamic parameters after a 8-week course of stimulation. mean ±SE (*n* = 5 SCI patients) **(A)** bladder capacity, **(B)** Voiding efficiency **(C)** baseline Pura prior to filling, **(D)** ΔP_ura_ during bladder filling, **(E)** ΔP_det_ during voiding as observed during clinical urodynamic studies at Pre-Therapy and Post-Therapy without TESCoN. mean ±SE (*n* = 5 stroke patients) **(F)** bladder capacity, **(G)** Voiding efficiency **(H)** baseline Pura prior to filling, **(I)** ΔP_ura_ during bladder filling, **(J)** ΔP_det_ during voiding as observed during clinical urodynamic studies at Pre-Therapy and Post-Therapy without TESCoN. ^∗^Significantly different from Pre-Therapy at *P* < *0.05.*

During baseline urodynamics, SCI patients demonstrated detrusor overactivity at low volumes, low voiding efficiency and detrusor sphincter dyssynergia during voiding. Stroke patients demonstrated low bladder capacity, detrusor overactivity, and appropriate voiding efficiency (Figure 2; Weld and Dmochowski, 2000; Weld et al., 2000). Acute delivery of TESCoN in SCI patients reduced detrusor overactivity, increased bladder capacity, improved coordination between detrusor and the external urethral sphincter and increased voiding efficiency (Figure 3), consistent with our earlier observations (Gad et al., 2018a). In contrast, stroke patients did not demonstrate a change in bladder capacity or voiding efficiency. However, stroke patients exhibited an increase in the volume at first bladder sensation, and a significant increase in the ability to delay urination, as measured by the time between reaching bladder capacity and initiation of voiding *(P* < *0.05)* (Figure 4). After completing the 8-week therapeutic intervention, both sets of patients (*n* = 5 Stroke and *n* = 5 SCI) demonstrated an increased bladder capacity (*P* < *0.05*, Figures 5A,F) (without TESCoN); however, no change in voiding efficiency was observed in either group (Figures 5B,G). The average baseline pressure recorded at the urethral port (P_ura_) and the change in P_ura_ during the filling phase of the urodynamic cycle were higher (*P* < *0.05*) after compared to before therapy (Figures 5C,D,H,I). Detrusor pressure (P_det_) during voiding did not change before vs. after therapy (Figures 5E,J).

### Clinical Assessment of Patients

**FIGURE 6:**
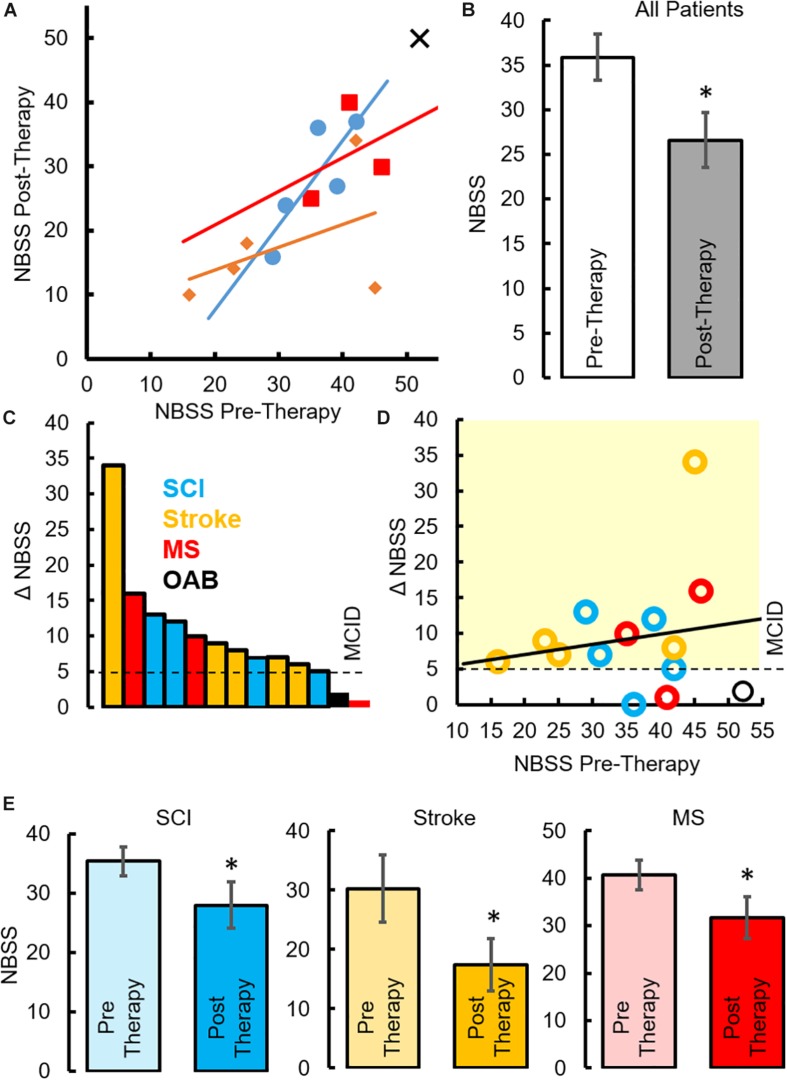
Changes in NBSS parameters after TESCoN therapy **(A)** Neurogenic Bladder Symptom Score (NBSS) at Pre-Therapy and Post-Therapy for the 14 patients tested. **(B)** Mean ± SE (*n* = 14 patients) NBSS scores at Pre-Therapy and Post-Therapy. **(C)** Distribution of NBSS score decrease across the 14 patients tested, note only 5 SCI patients are plotted since 5th patient observed a change of 0, **(D)** decrease in NBSS scores relative to the initial NBSS scores, **(E)** mean ±SE (*n* = 5 SCI patients, *n* = 5 stroke and *n* = 3 MS) NBSS scores at Pre-Therapy and Post-Therapy. MCID, minimal clinically important difference. ^∗^statistically significant from Pre-therapy at *P* < *0.05.*

**FIGURE 7:**
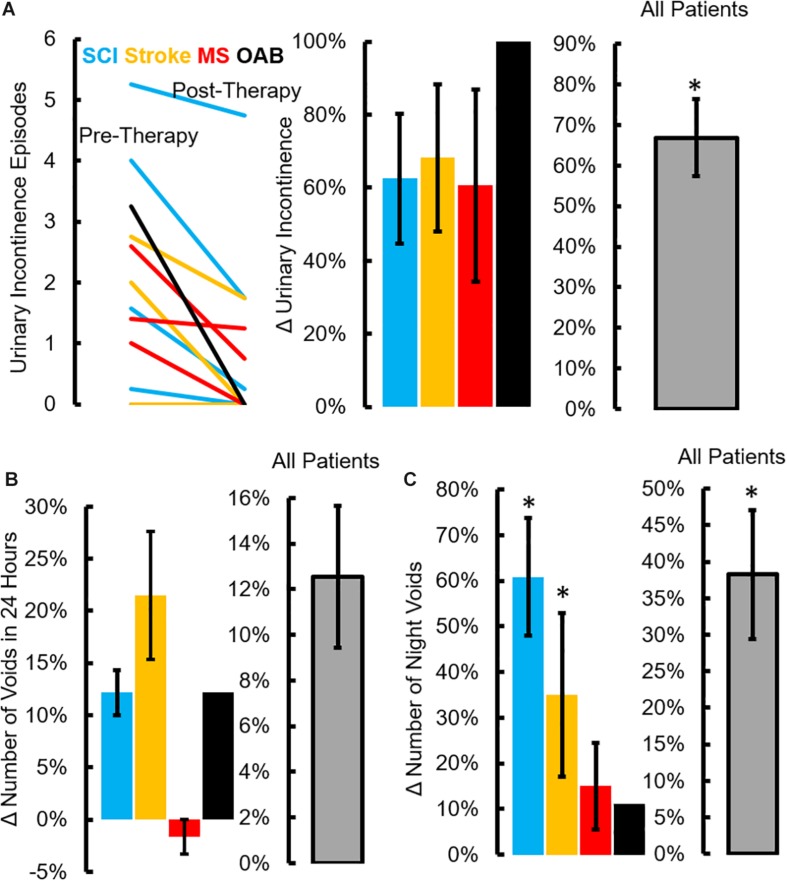
Changes in voiding diary parameters after TESCoN therapy. **(A)** Number of urinary incontinence at Pre-Therapy and Post-Therapy for the 14 patients tested, mean ± SE percent decrease in incontinence episodes for the 4 patient groups and all patients (*n* = 14 patients) tested, **(B)** mean ± SE percent decrease in number of voids for the 4 patient groups and all patients (*n* = 14 patients) tested and **(C)** mean ±SE percent decrease in number of night time voiding/CIC episodes during the night (10pm to 6am) for the 4 patient groups and all patients (*n* = 14 patients) tested. ^∗^Significanatly different from pretherapy at *P* < 0.05.

All patients underwent a clinical assessment in the form of a 4-day voiding diary and NBSS. Eleven (*n* = 4 SCI, *n* = 5 Stroke and *n* = 2 MS) out of thirteen neurogenic patients reported at least a five-point decrease (minimal clinically important difference, MCID) in the NBSS (Welk et al., 2018; Figure 6A). The mean score in the NBSS decreased from 35.9 ± 2.6 to 26.6 ± 3.1 *(P* < *0.05)* with the highest change being 34 points and the lowest being 0 (Figures 6C,D). Note that significant decrease in NBSS scores were observed in all pathologies (Figure 6E). The distribution of NBSS decrease among the different underlying pathologies showed no obvious trends. All patients also reported a significant decrease in the number of incontinence episodes/day (∼68% reduction in leaks, *P* < *0.05*) (Figure 7A), a reduction (12%) in the number of voiding/CIC episodes per day (Figure 7B) and a significant reduction (∼37%) in night time voiding/CIC episodes as recorded on the voiding diary (Figure 7C). No Adverse Events (AE) were reported. All patients reported to be satisfied with the therapy and would have continued beyond the 8 weeks if the therapy was offered.

## Discussion

### Neuromodulation Enables Restoration of Sensation and Motor Control of LUT

Multiple components of the nervous system play a role in LUT control. Thus, when one or more components of the nervous system are affected by a disease, LUT dysfunction can ensue. Although modern management techniques have ensured that LUT dysfunction is rarely dangerous, it almost inevitably has a marked impact on patients’ quality of life. Some current therapies for LUT dysfunction in neurogenic and idiopathic situations are effective for preventing incontinence (e.g., anticholinergics, beta-agonists, botulinum toxin injection) but they do not restore normal bladder sensation or voiding function and sometimes achieve continence at their expense. On the other hand, spinal neuromodulation is a technique whose premise is to restore neural control functions by delivering a sub-motor threshold electrical stimulus that can transform the controlling neural networks into more functional physiological states. After 8 weeks of non-invasive spinal cord stimulation, bladder capacity increased. SCI patients also demonstrated improved detrusor-sphincter dyssynergia during detrusor contractions. All patients reported an improved sensation of bladder fullness and an increased latency time between sensation of urgency and the first episode of detrusor overactivity (or volitional voiding contraction). These effects appeared to be durable as they were observed even 1 week after therapy was concluded.

A commons question in the field of neuromodulation is, how epidural and transcutaneous spinal stimulation compare? Some of the more Important comparisons are (1) the ability to deliver the desired stimulation parameters to the most functionally effective neuronal networks for a given target organ system, (2) patient acceptability and ease and accessibility of delivery of the intervention (3) cost of the intervention and (4) safety of the intervention. To date, there is insufficient knowledge to weigh the advantages, but it seems reasonably safe to conclude that both approaches should continue to be developed and tested. Given the data to date, it seems almost inevitable that the best choice of approach will be based on the net result of pros and cons for a given patient as judged by the patient, the physician and the caregivers.

To briefly expand on some of these points, it is obvious that using the transcutaneous approach, multiple organ systems can be targeted simply by moving the electrode along the length of the spinal cord (Gad et al., 2018a, b; Gad P. et al., 2018; Inanici et al., 2018; Phillips et al., 2018; Rath et al., 2018; Hofstoetter et al., 2019; Sayenko et al., 2019). Epidural stimulation has been effective in treating both autonomic (Harkema et al., 2018; Herrity et al., 2018; Hubscher et al., 2018) and motor functions (Grahn et al., 2017; Angeli et al., 2018; Gill et al., 2018; Wagner et al., 2018) while maintaining the overall location of the implant even though the scope of the neural networks being neuromodulated may be more limited compared to transcutaneous stimulation. Evidence to date suggest that the transcutaneous approach in general has a greater advantage because of a more encompassing combination of networks that can be modulated to multiple organ systems. The activation of a broader network may enable multiple muscle groups and rely of the automaticity and feedforwardness of the spinal cord (Gerasimenko et al., 2017). A disadvantage of the transcutaneous approach is the inconvenience to frequently don and doff the electrodes and the lower spatial resolution compared to the epidural approach. While only a side-by-side comparison in the same patient may provide more definitive answers, the transcutaneous approach definitely can help screen potential responders and provide insights regarding effective sights for stimulation. One could proceed from the transcutaneous approach to the Implantation strategy if this would be viewed as a more long-term solution. The reverse approach, however, would be more problematic.

### Clinically Significant Levels of LUT Function Can Be Restored

As important as the physiological changes observed on urodynamic testing were the clinical improvements assessed by the voiding diary and the NBSS. The NBSS is a validated questionnaire (Welk et al., 2018) that addresses common urological complaints in patients with neurological disease (e.g., incontinence, frequency, urgency and their impact on quality of life). The ability of NBSS to detect a meaningful clinical change has been recently shown in a pilot study of SCI and multiple sclerosis patients, receiving botulinum toxin injections for neurogenic urinary incontinence (Fragala et al., 2015). Over 85% of the neurogenic bladder patients in our study reported a statistically significant and clinically meaningful improvement in the overall NBSS score after TESCoN therapy was completed. Among the various domains of the questionnaire, there was improvement in the incidence of incontinence, quality of life and voiding/storage domains of the NBSS. As expected, there was no significant change in the consequences domain of the NBSS as the questions in this section of the questionnaire represent chronic problems related to the urinary tract (e.g., bladder and kidney stones), that would not be expected to improve immediately with positive change in LUT function. However, all patients also reported either a significant decrease in the number of urinary incontinence episodes per day or a decrease in the number of night time voiding cycles. Similar to the responses observed in the NBSS scores, no obvious trends were observed across the pathologies.

### Mechanistic Factors That Contribute to Restoration of LUT Functions

Although the mechanistic details of how spinal neuromodulation can improve bladder function is not known, multiple but highly linked mechanisms probably contribute to the observed improvements. We postulate that stimulation modulates both afferent and efferent spinal networks into a more functional state. Neuromodulation may alter the responsiveness of spinal networks to bladder filling and emptying and increase the conscious awareness of these states. After chronic TESCoN therapy, subjects reported improved bladder sensation and decreased urinary urgency. Together, these findings suggest that parts of the CNS responsible for conscious sensation in the brain may have been re-engaged along with re-activation and/or retraining of local spinal centers controlling the LUT, reflecting a highly significant level of functional neural plasticity. It is interesting to note that despite the varied pathology, location and severity of injury, the spinal control of detrusor and urethral sphincter muscles were intact and could be transformed using a non-invasive modality. Our finding that voiding efficiency after 8 weeks of treatments did not change when urodynamic test was repeated in absence TESCoN suggests that the parasympathetic system, that drives bladder emptying, may require ongoing stimulation in order to induce a functional change. On the other hand, it appears that TESCoN can induce long standing neuroplasticity in the sympathetic and somatic system, which drives bladder storage, as evidenced by our finding that bladder capacity and P_ura_ showed improvement even in the absence of stimulation.

Despite the potential shortcomings of the limited number of patients and lack of sham stimulation which will be addressed in future randomized controlled clinical trials, these data demonstrate the ability to transform the neural control of bladder function from a dysfunctional to a functional state using non-invasive spinal neuromodulation. Our previous results have demonstrated improvement in multiple functions (locomotion and autonomic function) with spinal neuromodulation during function rehabilitation (Gerasimenko et al., 2015; Gad et al., 2017; Gad P. et al., 2018). In this study, however, since the patients were seated while receiving TESCoN therapy, minimal improvements in locomotor function were observed. TESCoN could prove to be a critical component of in our clinical toolbox while designing rehabilitation therapies for patients that suffer from multiple organ dysfunction (autonomic and motor) due to paralysis. In addition, future studies will also identify potential chronic changes in the cortex during simultaneous functional MRI recordings during urodynamic studies. These data allow us to speculate about multiple neural mechanisms that can account for end-organ dysfunction in neurogenic and non-neurogenic states (e.g., loss of connectivity between centers responsible for end organ control, formation of aberrant neural connections resulting in abnormal function). However, the intrinsic spinal networks controlling the LUT seem to not only persist post injury, but also have the potential to undergo transformation to a more functional state. These observations are consistent with our studies of non-invasive spinal cord stimulation in other applications such as lower extremity (Gerasimenko et al., 2015; Gad et al., 2017), and upper extremity functional rehabilitation (Gad P. et al., 2018; Inanici et al., 2018), where we have consistently observed some restoration of voluntary control in individuals clinically diagnosed with complete motor and sensory paralysis. We hypothesize that neuromodulation enables activity-dependent mechanisms that transform functionally incompetent spinal and supraspinal networks to higher functional states. The idea that neuromodulation can affect a part of the CNS remote from the site of stimulation is supported by data from other groups. For example, sacral nerve stimulation (a peripheral nerve neuromodulation modality commonly employed for idiopathic overactive bladder) is known to generate changes in brain signaling even during acute delivery of stimulation (Dasgupta et al., 2005). The encouraging findings that TESCoN can improve LUT symptoms in a variety of disease states encourages the exploration of its use in other brain pathologies associated with LUT dysfunction (e.g., Parkinson’s disease, cerebral palsy), thus expanding the potential impact of this technology to a wider range of diseases.

## Conclusion

We have successfully demonstrated that TESCoN can (1) reduce detrusor overactivity, increase bladder capacity and reduce episodes of incontinence in patients with SCI, stroke, multiple sclerosis and idiopathic over active bladder, (2) functional transformation of the sensory component of bladder control to improve sensation of fullness bladder and awareness by delaying the time between reaching bladder capacity and initiation of voiding, and (3) significantly reduce the number of incontinence episodes and night time voids that also reflects in the changes in NBSS scores. These observations suggest that the level of functional autonomy that is intrinsic to the neural circuitry that controls bladder function. This is a highly attractive clinical target for regaining greater levels of function in SCI and other etiologies of neurogenic bladder because it is a non-invasive form of neuromodulation that can re-engage and restore, via activity-dependent mechanisms, the automaticity intrinsic to the autonomic control of the LUT.

## Data Availability Statement

All datasets generated for this study are included in the article/supplementary material.

## Ethics Statement

The studies involving human participants were reviewed and approved by the Institutional Review Board of Rancho Research Institute, the research arm of Rancho Los Amigos National Rehabilitation Center, Downey, CA, United States. The patients/participants provided their written informed consent to participate in this study.

## Author Contributions

EK, PG, and VE designed the study. EK performed the initial medical evaluation and performed the urodynamic study. HZ, KL, and SY performed the day to day stimulation. PG analyzed the data. EK and PG wrote the manuscript. All authors edited and approved the manuscript.

## Conflict of Interest

VE holds shareholder interest in NeuroRecovery Technologies and hold certain inventorship rights on intellectual property licensed by The Regents of the University of California to NeuroRecovery Technologies and its subsidiaries. VE and PG holds shareholder interest in spineX Inc., and hold certain inventorship rights on intellectual property licensed by The Regents of the University of California to spineX Inc. The remaining authors declare that the research was conducted in the absence of any commercial or financial relationships that could be construed as a potential conflict of interest.
